# On the origin of Acochlidia and other enigmatic euthyneuran gastropods, with implications for the systematics of Heterobranchia

**DOI:** 10.1186/1471-2148-10-323

**Published:** 2010-10-25

**Authors:** Katharina M Jörger, Isabella Stöger, Yasunori Kano, Hiroshi Fukuda, Thomas Knebelsberger, Michael Schrödl

**Affiliations:** 1 Bavarian State Collection of Zoology, Münchhausenstr. 21, 81247 München, Germany; 2 Department of Marine Ecosystems Dynamics, Atmosphere and Ocean Research Institute, University of Tokyo, 5-1-5 Kashiwanoha, Kashiwa, Chiba 277-8564, Japan; 3 Conservation of Aquatic Biodiversity, Faculty of Agriculture, Okayama University, Tsushima-naka 1-1-1, Kita-ku, Okayama 700-8530, Japan

## Abstract

**Background:**

A robust phylogenetic hypothesis of euthyneuran gastropods, as a basis to reconstructing their evolutionary history, is still hindered by several groups of aberrant, more or less worm-like slugs with unclear phylogenetic relationships. As a traditional "order" in the Opisthobranchia, the Acochlidia have a long history of controversial placements, among others influenced by convergent adaptation to the mainly meiofaunal habitats. The present study includes six out of seven acochlidian families in a comprehensive euthyneuran taxon sampling with special focus on minute, aberrant slugs. Since there is no fossil record of tiny, shell-less gastropods, a molecular clock was used to estimate divergence times within Euthyneura.

**Results:**

Our multi-locus molecular study confirms Acochlidia in a pulmonate relationship, as sister to Eupulmonata. Previous hypotheses of opisthobranch relations, or of a common origin with other meiofaunal Euthyneura, are clearly rejected. The enigmatic amphibious and insectivorous Aitengidae *incerta sedis *clusters within Acochlidia, as sister to meiofaunal and brackish Pseudunelidae and limnic Acochlidiidae. Euthyneura, Opisthobranchia and Pulmonata as traditionally defined are non-monophyletic. A relaxed molecular clock approach indicates a late Palaeozoic diversification of Euthyneura and a Mesozoic origin of the major euthyneuran diversity, including Acochlidia.

**Conclusions:**

The present study shows that the inclusion of small, enigmatic groups is necessary to solve deep-level phylogenetic relationships, and underlines that "pulmonate" and "opisthobranch" phylogeny, respectively, cannot be solved independently from each other. Our phylogenetic hypothesis requires reinvestigation of the traditional classification of Euthyneura: morphological synapomorphies of the traditionally defined Pulmonata and Opisthobranchia are evaluated in light of the presented phylogeny, and a redefinition of major groups is proposed. It is demonstrated that the invasion of the meiofaunal habitat has occurred several times independently in various euthyneuran taxa, leading to convergent adaptations previously misinterpreted as synapomorphies. The inclusion of Acochlidia extends the structural and biological diversity in pulmonates, presenting a remarkable flexibility concerning habitat choice.

## Background

Since the introduction of the Heterobranchia concept by Haszprunar [1,2], considerable advances have been achieved, solving the phylogeny of certain heterobranch groups (i.e. "families" or "orders") on morphological (e.g. Mikkelsen [3] on Cephalaspidea; Jensen [4] on Sacoglossa; Wägele and Willan [5] on Nudibranchia, Klussmann-Kolb [6] on Aplysiidae) and molecular levels (e.g. Wollscheid-Lengeling et al. [7] on Nudibranchia; Wade et al. [8] on Stylommatophora; Klussmann-Kolb and Dinapoli [9] on Pteropoda). Members of the Euthyneura - the major heterobranch clade - have conquered marine, limnic and terrestrial habitats from the deep sea to the high mountains. As a result they form one of the most successful and diverse groups within Gastropoda, and even within Mollusca as regards species numbers and ecological diversity. Quite some effort has been dedicated to revealing relationships in the taxon, and to supporting or rejecting the respective monophyly of traditional higher groupings such as Pulmonata and Opisthobranchia. Nevertheless, the phylogeny of the Euthyneura has remained partially unresolved and heavily discussed [see e.g. [10-17]]. While morphological analyses face the problem of convergent developments that might mask the true phylogenetic signal, and depend on the coding procedure for morphological characters [18], single-marker molecular analyses are challenged in choosing a suitable marker, and multi-locus molecular studies stand and fall with the available taxon sampling.

One major problem in molecular studies is that highly aberrant or derived taxa of uncertain taxonomic relationships "jump around" in phylogenetic analyses and weaken the phylogenetic signal for higher taxa. Members of such groups are often hard to obtain (especially for molecular purposes); thus, the groups are frequently either excluded from phylogenetic analyses or only included with a low number of representatives, resulting in poor overall taxon sampling. One attempt to support future phylogenetic approaches on a higher taxonomic level (i.e. Heterobranchia or Gastropoda) is to provide data on small enigmatic groups and their phylogenetic relationships step by step.

The Acochlidia, a traditional "order" of the Opisthobranchia since their establishment by Odhner [[19]; as Acochlidiacea], form one of the unsolved mysteries within Euthyneura [18]. Being a small group with only 28 valid species worldwide, these slugs are morphologically and biologically highly aberrant and diverse, comprising a series of unusual characters (e.g. secondary gonochorism, lack of copulatory organs, asymmetric radulae) [see e.g. [20-23]]. Most acochlidians live interstitially in marine sands, while some have conquered limnic systems (uniquely within opisthobranch gastropods). Their monophyly is widely accepted [20,22,24,25] especially since a proposed sister group relationship of the acochlidian family Ganitidae with Sacoglossa (based on the dagger-shaped radula teeth, see [26]) could be rejected based on a comprehensive parsimony analysis of morphological characters [22]. During the last years a series of studies have redescribed key acochlidian taxa in great detail, including 3D reconstructions [27-32], and added considerably to the morphological and biological knowledge of this previously little understood group. A first comprehensive cladistic analysis of their phylogeny is now established [22], but the identity of their sister group remains uncertain. Most recent morphological analyses suggested a common origin with either the equally enigmatic Rhodopemorpha [10], the diaphanid cephalaspidean *Toledonia *[25], or with runcinid or philinoid cephalaspideans [22,33]. However, morphology-based analyses by Schrödl and Neusser [22], demonstrated that Acochlidia usually group with other mesopsammic taxa, if any were included (i.e. with the sacoglossan *Platyhedyle*, the rhodopemorph *Rhodope *or the cephalaspideans *Philinoglossa *or *Philine exigua*). Thus, it is likely that convergent adaptations to the interstitial habitat mask the truly phylogenetic signals. Molecular markers independent from direct ecological pressures suggested an unresolved basal opisthobranch origin for Acochlidia ([34] based on nuclear 18S rRNA and 28S rRNA). A first combined multi-gene dataset led to the surprising result of Acochlidia clustering in a pulmonate relationship, united in a clade with Pyramidelloidea, Amphiboloidea and Eupulmonata [17]. However, only three derived acochlids [see [22]] were included, with partially missing data. Therefore this unexpected result requires re-examination based on complete multi-locus data and a more focused taxon sampling, including all previously suggested potential sister groups of Acochlidia. Most recently, another curiosity with potential affinities to Acochlidia has been described: the amphibious and insectivorous sea slug *Aiteng ater *from mangrove mud in Thailand [35]. Due to its unusual combination of morphological characters (prepharyngeal nerve ring, presence of ascus, uniseriate radula) it was placed in a new family, Aitengidae, with unclear phylogenetic relationships and affinities to Sacoglossa, Acochlidia and Cephalaspidea. A similar but still undescribed species was found in Japan, which was available for the present study. Morphologically it clearly belongs to the Aitengidae, but shows differences to *A. ater *at genus or species level (own unpublished data). Its affinity to *A. ater *is confirmed by comparison of the mitochondrial 16S rRNA-sequences (K. Händeler, pers. comm.).

The present study aims to clarify the origins and phylogenetic relationships of Acochlidia and potentially related enigmatic taxa such as Aitengidae, based on a combined molecular dataset from nuclear and mitochondrial markers. For the first time, representatives of six out of seven acochlidian families [22] are analysed in the context of a broad taxon sampling that includes other meiofaunal slugs (*Philinoglossa praelongata*, *Philine exigua*, *Smeagol phillipensis*) and most euthyneuran sub-groups. Furthermore, the potentially related *Gascoignella nukuli *(as a representative of Platyhedylidae) and an undescribed species of Aitengidae are included in the present study. Since there is no fossil record of Acochlidia or any other mesopsammic Euthyneura, we apply a molecular clock approach to estimate divergence times for these groups. On the basis of our phylogenetic hypothesis we discuss evolutionary trends and potential consequences for euthyneuran classification in general.

## Results

### Neighbournet analysis

The neighbournet graph created by SplitsTree 4 (see Additional File 1) visualises a generally high conflict in the data (shown by a netlike structure with edges of similar length), and high substitution rates displayed by long terminal branches in many taxa. There is no clade-supporting pattern for the monophyly of Opisthobranchia or of Pulmonata on the basis of our dataset. Of the major traditional heterobranch taxa only Acteonoidea and Nudipleura show a clear split support (visualised by long parallel edges); some split support is present for Pyramidelloidea, Cephalaspidea s.s., Anaspidea, Umbraculoidea, pteropod Gymnosomata and Thecosomata, Amphiboloidea and Siphonarioidea. No pattern supporting any of the other opisthobranch or pulmonate groups can be found, mainly due to affinities of individual species to neighbouring groups. No split pattern indicates a relationship between the different meiofaunal heterobranchs such as Acochlidia, *Smeagol phillipensis *and Philinoidea (*Philinoglossa praelongata *and *Philine exigua*) (see Additional File 1).

The monophyly of the Acochlidia receives no split support. A very weak signal supports a grouping of Acochlidia together with some pulmonate taxa, but there is no indication for affinities to other opisthobranch taxa. The acochlidian subgroups Hedylopsacea and Microhedylacea receive no split support, due to some common support for *Hedylopsis *(Hedylopsacea) and *Asperspina *(Microhedylacea). The enigmatic Aitengidae sp. receives split support grouped with acochlidian Pseudunelidae and Acochlidiidae, and shows no affinity to Sacoglossa or Cephalaspidea.

### Phylogenetic analysis

**Figure 1:**
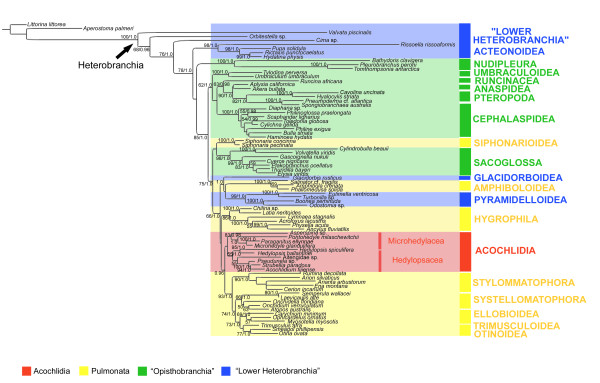
**Origin of Acochlidia within Heterobranchia**. RAxML analysis based on the combined dataset (18S, 28S, 16S, COI), masked with Gblocks. Bootstrap support and posterior probabilities given (only BS ≥ 50 and PP ≥ 0.95 are considered relevant and thus shown).

Examination of differences in incongruence length between the four genetic markers - 18S rRNA, 28S rRNA, 16S rRNA and cytochrome *c *oxidase subunit I (COI) - using the ILD-test implemented in PAUP* [36] revealed that the phylogenetic signal is improved in the combined data set (p-value of 0.01). Thus a concatenated dataset was used for phylogenetic analyses. The likelihood values of the different partitions of the dataset were compared via the Akaike Information Criterion (AIC) and the separation into 5 partitions (one each for 18S, 28S and 16S; COI separated in the two partitions 1^st ^and 2^nd ^position and 3^rd ^position) improved the likelihood significantly (see Additional File 2). The dataset aligned with MAFFT, masked with Gblocks and analysed in 5 partitions led to the best likelihood value, thus it is presented herein as the most probable phylogenetic hypothesis based on our data (see Figure 1). For comparison of the different analytical approaches and the resulting differences in tree topology and related support values, see Table 1.

**Table 1:** Summary of the different analyses conducted

Sequence alignment/masking/phylogenetic analysis	Model of sequence evolution	Length of alignment	Monophyly of Acochlidia and sister group relationship	Changes within the tree topology compared to Figure 1
MAFFT + Gblocks + RAxML	GTRCAT GTR + GAMMA	3641 bp	see Figure 1	see Figure 1
MAFFT + ALISCORE + RAxML	GTRCAT GTR + GAMMA	3926 bp	Acochlidia monophyletic (no BS support) Aitengidae basal within Hedylopsacea; Acochlidia sister to (Hygrophila + (Glacidorboidea + (Amphiboloidea + Pyramidelloidea))) (no BS support)	Anaspidea non-monophyletic; different internal topology of Cephalaspidea s.s. (Philine exigua basal to remaining taxa); Siphonarioidea and Sacoglossa form no clade, but Siphonarioidea + (Sacoglossa + remaining pulmonate taxa)
MAFFT + Gblocks + MrBayes	GTR + G + I	3641 bp	Acochlidia monophyletic (no significant PP); sister group to Eupulmonata (0.96 PP)	basal tritomy within Euthyneura: (Acteonoidea + Rissoelloidea)/Nudipleura/remaining Euthyneura; different internal topology of Cephalaspidea s.s. (Philine exigua basal to remaining taxa), ((Glacidorboidea + Amphiboloidea) + Pyramidelloidea)
MAFFT + ALISCORE + MrBayes	GTR + G + I	3926 bp	Acochlidia monophyletic (no significant PP) Aitengidae basal within Hedylopsacea; Acochlidia sister to (Hygrophila + (Glacidorboidea + Amphiboloidea + Pyramidelloidea)) (no significant PP)	Anaspidea non-monophyletic; different internal topology of Cephalaspidea s.s. (Philine exigua basal to remaining taxa); Siphonarioidea and Sacoglossa form no clade, but Siphonarioidea + (Sacoglossa + remaining pulmonate taxa); Nudipleura form a basal clade with (Acteonoidea + Rissoelloidea)

The table lists the different methods of masking the alignment, phylogenetic approaches and models of sequence evolution used for the different analyses, as well as the resulting differences in tree topology (bootstrap support = BS; posterior probability = PP).

The Euthyneura form a monophyletic group without significant bootstrap support (BS) in ML-analyses, or posterior probability (PP) in Bayesian analyses. They do not include the Acteonoidea (sister to "lower heterobranch" Rissoelloidea) in most of our analyses, but include the Pyramidelloidea and Glacidorboidea as sister group to Amphiboloidea. Within the Euthyneura the Opisthobranchia clearly result as non-monophyletic. At the basis of the Euthyneura the Nudipleura split off, with high internal support. The clade of the remaining euthyneuran taxa receives good support (85 BS/1.0 PP). First, an opisthobranch clade (no significant BS/1.0) is composed of Umbraculoidea, Runcinacea, Cephalaspidea s.s., Anaspidea and Pteropoda, with Umbraculoidea as the most basal branch. The runcinid *Runcina africana *forms the sister group to the Anaspidea and the well backed (82/1.0) Pteropoda (Gymnosomata and Thecosomata), and the above combined are sister to the remaining Cephalaspidea s.s., with high support for monophyly of Cephalaspidea s.s. (100/1.0). Internally the Cephalaspidea s.s. are poorly resolved, and their internal topology differs between the RAxML and Bayesian analyses (see Table 1). The mesopsammic *Philine exigua *and *Philinoglossa praelongata *do not form a clade: *P. praelongata *clusters with *Scaphander lignarius*, whereas no clear sister group relationship could be identified for *P. exigua*.

The Pulmonata as traditionally defined result as non-monophyletic due to the inclusion of the opisthobranch groups Sacoglossa and Acochlidia and of the "lower" heterobranch Pyramidelloidea and Glacidorboidea. The pulmonate clade is significantly supported (75/1.0), but internally characterised by an unstable topology, with no or low support concerning the sister group relationships between the major groups. Siphonarioidea and Sacoglossa form a clade (lacking significant support) sister to the remaining taxa (see Figure 1). In the analyses of the ALISCORE dataset Siphonarioidea form the most basal group, followed by a split-off of the Sacoglossa (see Table 1). The monophyletic Sacoglossa (98/1.0) combine clades with shelled and shell-less representatives, with *Gascoignella nukuli *(Platyhedylidae) as the most basal offshoot of the latter. Siphonarioidea + Sacoglossa are recovered as sister group to a clade composed of (Glacidorboidea + (Amphiboloidea + Pyramidelloidea)) + (Hygrophila + (Eupulmonata + Acochlidia)). Apart from Acochlidia, the monophyly of all higher taxa is well supported: Amphiboloidea (100/1.0), Pyramidelloidea (99/1.0), Hygrophila (86/1.0) and Eupulmonata (93/1.0). However, relations between these taxa are poorly resolved, not supported, and vary within the different analyses (see Table 1). In all our analyses Amphiboloidea cluster with Glacidorboidea and Pyramidelloidea. Thus Thalassophila (= Siphonarioidea and Amphiboloidea) and Basommatophora (= Thalassophila and Hygrophila) are left as polyphyletic. The Eupulmonata (Stylommatophora, Systellommatophora, Ellobioidea, Trimusculoidea and Otinoidea) are recovered sister to Acochlidia. Within Eupulmonata Stylommatophora (90/1.0) form the basal group; Systellommatophora (no significant BS/1.0) is sister to a clade Ellobioidea + (Trimusculoidea + Otinoidea), the latter comprising *Smeagol phillippensis *and *Otina ovata*.

Acochlidia are recovered as monophyletic but with no significant support. The internal phylogeny of the Acochlidia is composed of the two monophyletic traditional suborders Hedylopsacea (with Hedylopsidae, Pseudunelidae and Acochlidiidae) and Microhedylacea (with Asperspinidae and Microhedylidae including Ganitidae), and is congruent with the morphology-based phylogeny of Acochlidia proposed by Schrödl and Neusser [22]. Additionally the enigmatic Aitengidae sp. clusters within the Hedylopsacea as sister group to Pseudunelidae and Acochlidiidae (see Figure 1) or basal within Hedylopsacea.

In analyses of Gblock datasets Acochlidia are sister to Eupulmonata (see Figure 1), in ALISCORE based analyses they cluster sister to Hygrophila + (Glacidorboidea + Amphiboloidea + Pyramidelloidea) (see Table 1). To assess the level of confidence of the "best" tree (i.e. pulmonate relationship of Acochlidia), we calculated the p-values of an alternative topology (Acochlidia cluster within Opisthobranchia) in combination with the "best" tree topology. Based on the resulting p-values of the AU test the alternative hypothesis is highly significantly rejected (AU value = 0).

### Molecular clock

**Figure 2:**
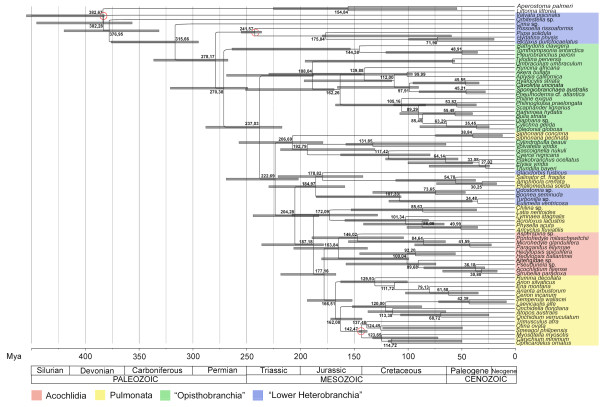
**Chronogram of Heterobranchia**. Showing estimated divergence times obtained from BEAST v1.5.3 under the uncorrelated lognormal relaxed clock model. Numbers at nodes refer to node ages in this presented tree (maximum-clade-credibility-tree); bars express 95% highest posterior density (HPD) (only presented for nodes with a PP > 0.5). Red circles indicate calibration points. Geological timescale is based on the International Stratigraphic Chart by the International Commission on Stratigraphy (2009).

The phylogenetic hypothesis obtained with the software BEAST (see Figure 2) based on the concatenated four-marker Gblocks dataset largely confirms the topology obtained from RAxML and MrBayes (see Figure 1). Based on the three fossil calibration points the Euthyneura originated already in the Palaeozoic, probably in the Carboniferous or Permian. The diversification of Euthyneura with the rise of many extant taxa started approximately in the late Palaeozoic (Permian) and major divergence events occurred in the Mesozoic. On the basis of our analysis the pulmonate clade (also including Sacoglossa, Acochlidia, Pyramidelloidea and Glacidorboidea) first appeared in the late Palaeozoic to early Mesozoic, approximately at the Permian/Triassic transition. The split between Eupulmonata and Acochlidia took place in the Mesozoic, between the Triassic and Jurassic periods. The diversification of Acochlidia is estimated to have happened in the Jurassic with the split between Hedylopsacea and Microhedylacea. Aitengidae split off from Pseudunelidae and Acochlidiidae in the Cretaceous. The transition to limnic habitats within Acochlidiidae appears as a comparatively recent event dating to the Palaeogene.

According to our data, major opisthobranch groups originated also in the Mesozoic (e.g. Cephalaspidea s.s. estimated to the Jurassic, Sacoglossa approximately Triassic/early Jurassic period, Pteropoda to the Cretaceous).

For comparison and to evaluate the impact of removing ambiguous parts of the alignment on molecular clock analyses, we repeated the analysis with the raw (i.e. uncut) alignment of our data (again using the concatenated four-marker dataset in five partitions). Even though the topology varied slightly from the one in the previous analysis, the estimated divergence times stayed surprisingly constant, supporting the rough estimate given above.

## Discussion

### Implications for the phylogeny of Heterobranchia

Our results on the origin of Acochlidia - in congruence with previous molecular studies on Euthyneura based on the same molecular markers [14,17] - necessitate the reconsideration of current classification concepts. Redefinitions below aim to observe continuity in traditional nomenclature and cause the unavoidable minimum of changes in terminology.

#### Euthyneura

The monophyly of Euthyneura (traditionally uniting Opisthobranchia and Pulmonata) has been widely accepted and well supported [13,18,37], even though their eponymous apomorphy - the euthyneury - has been revealed as convergent development [1,2]. Euthyneuran monophyly was recently questioned due to inclusion of "lower Heterobranchia" Pyramidelloidea unresolved within Pulmonata [13,15,16] or sister to Amphiboloidea [14,17]. Some other morphological studies place Pyramidelloidea as sister to Euthyneura [10,33]. Dinapoli and Klussmann-Kolb [14] argued to include them within Euthyneura, which has also been supported by morphological analysis [13]. Latest molecular data on Pyramidelloidea support an euthyneuran origin and indicate a relationship with Glacidorboidea and Amphiboloidea [38]. Our data again recovers Pyramidelloidea as sister to Amphiboloidea within pulmonates (see Figure 1), but with no significant support. In addition to nucleotide sequences [[14,15,17], present study], data from mitochondrial gene arrangements [16], a "morpho-molecular" synapomorphy (20 bp deletion in 16S rRNA helix of Pyramidelloidea and Euthyneura, see [11]) as well as morphology (presence of a euthyneurous nervous system with giant nerve cells) all support the inclusion of Pyramidelloidea within Euthyneura. When first describing Glacidorboidea, Ponder [39] placed them within Pulmonata and discussed a relationship to Amphiboloidea. However, Haszprunar [2] moved them to "lower Heterobranchia". The first molecular data on Glacidorboidea confirmed a pulmonate relationship [14]. This is again supported by our data.

#### "Opisthobranchia"

While the monophyly of several opisthobranch subgroups (e.g. Pteropoda, Cephalaspidea s.s., Nudipleura) receives good support, the monophyly of the Opisthobranchia in a traditional sense is rejected in all recent studies, regardless of whether the latter are molecular or morphological [e.g. [14,17,40]]. This is confirmed by our multi-locus molecular approach (see Figure 1) and supported by the results of the AU test. Thus, "Opisthobranchia" as traditionally defined should be considered as non-monophyletic.

As in previous studies we can clearly distinguish at least two clades (i.e. basal Nudipleura and Umbraculoidea + Runcinacea + Anaspidea + Pteropoda + Cephalaspidea s.s.) within "Opisthobranchia" that lead towards the pulmonate level of organisation.

Only one of our analyses indicates the Acteonoidea sister to Nudipleura (see Table 1). This clade that had resulted repeatedly in molecular studies with still limited "lower heterobranch" taxon sampling, either in a derived position [34,41] or as a basal offshoot within Euthyneura [15,17]. A recent molecular phylogeny on Acteonoidea suggest a common origin with lower heterobranch Rissoelloidea and a sister group relationship to Nudipleura [42]. While the basal position of Acteonoidea was commonly accepted [33,40], some authors doubted the basal position of Nudipleura, which was originally considered as a highly derived taxon, and suspect rate heterogeneity and deviant base composition as causing this unnatural grouping [17,34]. Based on potential synapomorphies in the reproductive system (presence of a ciliary stripe within the ampulla, androdiaulic or triaulic pallial gonoduct), Ghiselin [43] already suggested a relationship between Acteonoidea and Nudipleura. However, Acteonoidea form a well-supported "lower heterobranch" clade with Rissoelloidea, (see Figure 1; Table 1), confirming results by Aktipis et al. [44] and Dinapoli and Klussmann-Kolb [14]. The latter authors also recovered Nudipleura as the first offshoot of Euthyneura, which is confirmed by our study. Salvini-Plawen and Steiner [10] grouped Umbraculoidea with Nudipleura, but none of the recent molecular or morphological studies support such a relationship [17,33,34].

A common clade including Umbraculoidea, Anaspidea, Cephalaspidea s.s. and Pteropoda was already well supported in previous molecular analyses [9,14,17], and monophyly of a clade Anaspidea + Pteropoda received strong support in one previous study [12]. The present results confirm Cephalaspidea s.s., including Diaphanidae, but excluding Runcinidae as suggested in a previous analysis [45]. In our study *Runcina africana *groups with Anaspidea and Pteropoda, as in the Bayesian analysis of the concatenated 18S rRNA, 28S rRNA and COI dataset of the more comprehensive cephalaspidean phylogeny by Malaquias et al. [45]. The latter authors thus proposed to reinstate Runcinacea as a taxonomic category equivalent to Cephalaspidea s.s.. However, different analyses of the same authors led to different placements of Runcinacea, e.g. as sister to the remaining Cephalaspidea s.s.; hence the group's origin was left unresolved. Surprisingly our study indicates independent origins for the mesopsammic *Philine exigua *(Philinidae) and *Philinoglossa praelongata*(Philinoglossidae). The internal topology of Cephalaspidea s.s. is weakly supported in our study, but a more complete cephalaspidean sampling also rendered Philinoidea paraphyletic (based on 18S and 28S) [45].

**Figure 3:**
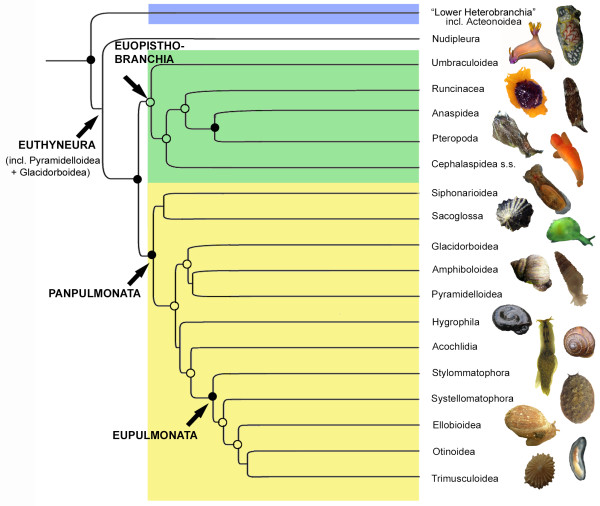
**Proposed reclassification of Euthyneura, discussed groups shown**. Nodes with significant support (i.e. > 75% bootstrap support (BS) and > 0.95 posterior probability (PP)) marked by dots; nodes with > 0.95 PP but low BS marked by circles. (Note: "Lower Heterobranchia" does not form a clade in the present study (see Figure 1), the branches are collapsed in the present Figure for illustration purposes.)

Based on our results and in congruence with the topology in previous studies [14,17], we suggest to unite Umbraculoidea, Anaspidea, Runcinacea, Pteropoda and Cephalaspidea s.s. in the new clade Euopisthobranchia (see Figure 3), presenting a monophyletic remainder of the "Opisthobranchia" as traditionally defined. Previous studies [9,18] discussed the gizzard (i.e. a muscular oesophageal crop lined with cuticula) with gizzard plates as homologous apomorphic structures supporting a clade composed of Cephalaspidea s.s., Pteropoda and Anaspidea. A gizzard with gizzard plates probably originated in herbivorous taxa in which it worked like a grinding mill, thus might be secondarily reduced in carnivorous groups within Cephalaspidea s.s. and Gymnosomata [9]. Klussmann-Kolb and Dinapoli [9] considered the gizzard in Umbraculoidea as non-homologous with the one in the previous groups, on account of the absence of gizzard plates or spines. This contradicted Salvini-Plawen and Steiner [10], who had proposed the gizzard to be a synapomorphy of the larger clade of Paratectibranchia (Pteropoda, Cephalaspidea and Anaspidea) and Eleutherobranchia, secondarily lost in Nudipleura but still present in Umbraculoidea. As coded in Wägele and Klussmann-Kolb [33], our phylogenetic hypothesis supports homology of the gizzard in Umbraculoidea with the gizzard with gizzard plates and spines in the other euopisthobranchian taxa. Thus, the structure is proposed as a synapomorphy of Euopisthobranchia.

#### "Pulmonata"

The monophyly of Pulmonata as traditionally defined has been well supported in morphological analyses (see e.g. [10,13]) and molecular studies [8,46]. However, doubts have arisen recently due to molecular studies which recovered additional taxa (e.g. Pyramidelloidea, Sacoglossa or Acochlidia) within "Pulmonata" [14,17], or to novel studies based on mitochondrial gene arrangements [16] which rendered "Pulmonata" polyphyletic. Based on our phylogenetic hypothesis (Figure 1) "Pulmonata" as traditionally defined is non-monophyletic due to the inclusion of Pyramidelloidea, Glacidorboidea, Sacoglossa and Acochlidia. On the premise of monophyletic Euthyneura, with basal Nudipleura and monophyletic Euopisthobranchia (see discussion above), the remaining euthyneuran taxa necessarily form a clade, in our study supported with maximum posterior probability (1.0) and significant bootstrap support (75%) (see Figure 1). Even though the topology within this pulmonate clade is unstable and not well resolved (see Table1), for practical reasons and due to the assumptions of monophyletic Euthyneura and Euopisthobranchia we suggest the new taxon Panpulmonata to unite Siphonarioidea, Sacoglossa, Glacidorboidea, Pyramidelloidea, Amphiboloidea, Hygrophila, Acochlidia and Eupulmonata (see Figure 3). The scientific meaning of the name "Pulmonata" and the corresponding major feature of those animals being "air-breathers" surely are not applicable to the novel panpulmonate groups Acochlidia, Sacoglossa and Pyramidelloidea, but also not for traditional pulmonate taxa such as Siphonarioidea or Hygrophila, most members of which lack permanently air-filled lungs. The term Panpulmonata is chosen for continuity in terminology. While certain pulmonate groups are well supported morphologically and molecularly (i.e. Eupulmonata and Hygrophila), unambiguous synapomorphies for Panpulmonata are hard to find (see discussion below).

Siphonarioidea and Sacoglossa form a clade sister to the remaining Panpulmonata (see Figure 3). While Haller [47] classified Siphonarioidea as opisthobranchs (e.g. on account of the presence of a gill), nowadays they are usually considered as "primitive" pulmonates, either grouped at the basis of the remaining Pulmonata [37,46] or united with Amphiboloidea as basommatophoran Thalassophila [48]. Molecular studies rendered Basommatophora and Thalassophila paraphyletic and indicated a close relationship of Siphonarioidea to Sacoglossa, either both within Opisthobranchia [16], at their basis [15], or basal to the remaining Pulmonata [[14,17], present study] as sister groups or separate clades. However, all studies show weak support at these nodes, and the positions of siphonariids and sacoglossans as well as their relationship still need confirmation by other character sets and improved taxon sampling.

In the present study the monophyly of Sacoglossa is well supported and also the split into shelled Oxynoacea and Plakobranchacea is well backed (see Figure 1). Both suborders are also well supported morphologically [4]. Platyhedylidae stand basally within the latter, as sister to Limapontioidea plus the remaining Plakobranchoidea. Jensen [4] placed Platyhedylidae at the basis of Plakobranchoidea but already pointed out their unclear relationships.

Hygrophila, Amphiboloidea and Eupulmonata are all well supported monophyletic groups in the present study, but their sister group relationships are not well resolved and receive little to no support.

### Origin of Acochlidia

All groups previously discussed as having an affinity or closer relationship to Acochlidia were included in the present study to reveal their phylogenetic relationships. Only the enigmatic Rhodopemorpha are lacking, but a recent molecular phylogeny based on nuclear and mitochondrial markers shows no affinities between Acochlidia and Rhodopemorpha [49], and the morphological characters common to both groups can be explained as convergent developments (see discussion below and [22]). A phylogenetic relationship of Acochlidia with the diaphanid *Toledonia*, which was suggested based on similar radula characteristics [25], is rejected by the present molecular data and also resulting from morphological analyses [22]. Morphological studies indicated a common origin for small Runcinacea and Cephalaspidea (i.e. mesopsammic *Philinoglossa *and *Philine exigua*) with Acochlidia [22,33]. However, Schrödl and Neusser [22] showed the liability of the topology to inclusion of other interstitial taxa such as *Rhodope *and *Platyhedyle*, which always resulted as direct sister groups to Acochlidia in various analyses. The authors thus concluded that the convergent adaptations to the interstitial habitat (e.g. worm-shaped body, development of spicules, loss of pigmentation) mask the true phylogenetic signal. This interpretation is supported by our SplitsTree analysis (see Additional File 1) and the present molecular results (see Figure 1), which clearly signal independent evolutionary origins for all the different mesopsammic Heterobranchia included here.

Previous molecular analyses placed the Acochlidia basally in an unresolved opisthobranch level [34] or surprisingly clustered them in an unresolved pulmonate relationship [17]. While any opisthobranch affinities are rejected based on split support (see Additional File 1), based on the AU test and based on phylogenetic analysis, the pulmonate relationship of Acochlidia is confirmed in this study (see Figure 1), which presents a much better acochlidian taxon sampling and highly likely topology within Acochlidia (see discussion below). Even though support for their direct sister group relationships are low and the topology varies between the different analyses, all analyses performed in the present study placed Acochlidia within pulmonates (see Table 1). This grouping based on molecular markers requires a re-evaluation of morphological characters and earlier, potentially biased homology assumptions, and a search for potential synapomorphies uniting Acochlidia with pulmonates. Three anatomical characters are generally accepted as true synapomorphies of the "Pulmonata" as traditionally defined: the pallial cavity opening by means of a pneumostome, presence of a procerebrum (with cerebral gland and double cerebro-connectives) and the existence of medio-dorsal (cerebral) bodies [13,50].

#### 1) Pallial cavity opening by means of a pneumostome

Although denied by some earlier authors, the pulmonary cavity of "Pulmonata" is today generally considered as homologous to the pallial cavity of non-pulmonate gastropods [51]. Whereas the loss of a gill and the presence of a "lung" certainly is a matter of multiple convergence paralleled in several prosobranch clades, the acquisition of a pneumostome (i.e. a small respiratory opening) is considered as synapomorphic for "Pulmonata" [13,18,48]. Dayrat and Tillier [[13], see also references therein] pointed out that the pneumostome of Siphonarioidea is not contractile, and their phylogenetic hypothesis [13] favoured homology with the pneumostome of the remaining Pulmonata. On the other hand, at least some siphonariids are reported to open and close their pneumostome [e.g. [52]]. A morphocline from a wide open pallial cavity to a narrow, nearly closed one (i.e. presence of pneumostome) is present in both "Opisthobranchia" and "Pulmonata"; thus the presence of a pneumostome in general cannot be considered as a pulmonate synapomorphy [53]. Barker [53] also questioned the synapomorphic contractile pneumostome, which might have evolved independently in different pulmonate taxa, e.g. in Eupulmonata and some Siphonarioidea. The presence of a small opening seems to be variable, indeed, and might depend on the habitat. For example, the truly subtidal marine *Williamia *(Siphonarioidea) have a wide opening [54], while intertial *Siphonaria *have a small one (i.e. a contractile or non-contractile pneumostome). The opening is wide also in subtidal shell-bearing Sacoglossa [3], whereas the pallial cavity is usually reduced in shell-less Sacoglossa. Pyramidelloidea also have a wide opening. In general within "Pulmonata" the "lung" undergoes a series of reductions; e.g., the tiny *Smeagol climoi *only has a small pallial cavity without respiratory function [51], as do larger Onchidiidae. A small, reduced pallial cavity can still be found in the quite basal acochlidian *Hedylopsis ballantinei *[55] (as *Hedylopsis *sp.), while all remaining Acochlidia studied so far entirely lack such a cavity [22,30]. All hedylopsacean nervous systems described in detail contain an osphradial ganglion [25,29,31,32], which can be interpreted as a remainder of an osphradium that was reduced in the course of the reduction of the pallial cavity. A group of derived, benthic and limnic acochlidians have developed a sensory, osphradium-like organ [56] like the one reported for the basal ellobiid *Ovatella *[57].

#### 2) Presence of a procerebrum

The procerebrum of "Pulmonata" is defined as an accessory lobe linked to the cerebral ganglion via two connectives, associated to the optic, tentacular and peritentacular nerves [58]. Its homology with the opisthobranch rhinophoral ganglion has long been discussed [2,47,59]. The configuration of the cerebral nerves and associated ganglia is complex in Acochlidia. The labiotentacular nerve arises ventrally from the cerebral ganglion; the rhinophoral ganglion usually gives rise to the rhinophoral nerve (with Hancock's nerve branching off), and the optic ganglion to the optic nerve ([31,32,56] and own unpublished data). However, in *Pseudunela cornuta *the optic nerve splits off from the rhinophoral nerve, and no nerves arise from the optic ganglion [29]. A similar arrangement occurs in *Hedylopsis spiculifera *and *H. ballantinei*, except that the optic ganglion is lacking [25,60]. In the microhedylaceans *Pontohedyle *and *Microhedyle *the rhinophoral nerve emerges directly from the cerebral ganglion, and eyes nestle directly on it ([27], own unpublished data); thus the additional ganglion might refer to either the rhinophoral or the optic ganglion. Tillier et al. [46] discussed a potential homology between the optic ganglion in "Opisthobranchia" and the pulmonate procerebrum. In Acochlidia double cerebral connectives could be identified for the rhinophoral ganglion in *Tantulum elegans*[60], the optic (but not the rhinophoral) in *Strubellia paradoxa *[56], and for the unclear optic/rhinophoral ganglion in *Pontohedyle milaschewitchii *and *Microhedyle glandulifera *([27] as rhinophoral ganglion, own unpublished data). The variable development of cerebral features in Acochlidia makes homologisation difficult at this time. Rhinophoral and optic ganglia are closely related to and might develop from the cerebral ganglion, and they share common features with the pulmonate procerebrum. Based on our phylogenetic hypothesis, the plesiomorphic state for Panpulmonata might be separate rhinophoral and optic ganglia that have been fused various times independently. However, the presence of so-called "globineurons" - neurons with densely packed, small, round nuclei - in Eupulmonata [58,61] appears to be a synapomorphy for this clade.

Additionally, the presence of a cerebral gland - a small, tube-like structure involved in the formation of the procerebrum - is considered as characteristic for the pulmonate nervous system [58,61]. This ectodermal structure may form a tube-like process from the procerebrum towards the lateral head region, or it may be reduced to a small epithelial cavity attached or enclosed within the procerebrum [58,61]. No structure similar to the cerebral gland has been described for Acochlidia, but due to the small size of the cerebral gland and the previously unknown pulmonate affinities of Acochlidia it might have been overlooked in morphological studies; hence, ultrastructural reinvestigations of acochlidian nervous systems are needed in the future. The cerebral gland is lacking also in other pulmonate taxa, e.g. Amphiboloidea [58], which either raises doubts about their pulmonate affinities [46] or suggests that the structure might have been lost secondarily. Moreover, Tardy [62,63] described a similar invagination involved in the formation of the rhinophoral ganglion in different nudibranchs. In light of the present phylogenetic hypothesis, with Nudipleura as the most basal euthyneuran offshoot, this might indicate that the formation of the rhinophoral ganglion (and the homologous procerebrum) involving an ectodermal invagination is plesiomorphic within Euthyneura, and that there are remnants (or paedomorphotic reinstatements) of this structure in adults of (some) pulmonate taxa.

#### 3) Presence of medio-dorsal (= cerebral) bodies

(Medio-)dorsal bodies (also termed cerebral bodies) are endocrine organs situated dorsally of the cerebral ganglia in "Pulmonata" [13], but considerable variation exists within the main pulmonate groups as regards the structure and innervation of the dorsal bodies [58,61,64]. Similar structures closely attached to the cerebral ganglia have been found in several Acochlidia: First described as "dorsal bodies" [25], they were later renamed "lateral bodies" by Neusser et al. [60], due to their more lateral position to the central nervous system and the unclear homology to pulmonate dorsal bodies. Since dorsal bodies in Pulmonata play a role in female reproduction [64], they might be fully developed in female adults only, thus might have been overlooked in some studies of gonochoristic acochlidian species or of hermaphrodites with "sex change". Further ultrastructural data on acochlidian "lateral bodies" and their potentially neurosecretory function are needed to evaluate homology with pulmonate structures. Moreover, pulmonate dorsal bodies might be homologous to the juxtaganglionar organs of some opisthobranchs [60], and thus might represent a plesiomorphic character of Panpulmonata and a potential synapomorphy of Euthyneura.

In addition, the presence of an unpaired dorsal jaw, which probably originated through the fusion of the paired lateral jaws [65], has been discussed as a potential synapomorphy of "Pulmonata" [18,48]. The presence of a pair of dorso-lateral jaws is a plesiomorphic character state for Euthyneura [13,65], but that condition has been reduced various times independently in "Opisthobranchia" and "Pulmonata" [18]. A dorsal, unpaired jaw might have evolved at the basis of Panpulmonata, and then have been secondarily reduced various times independently (e.g. in Onchidiidae, *Amphibola*) [18]. In Acochlidia, jaw-like structures are reported only for the derived microhedylacean family Ganitidae (as paired jaws), and as unclear "cuticular elements" for *Microhedyle glandulifera *(see [22] for citations). According to the derived position of Ganitidae in morphological [22] and molecular analyses (present study), these structures may represent either secondary developments (potentially related to the specialised dagger-shaped radula) or paedomorphic structures; however, studies of Acochlidia larvae are still overdue.

The only potential synapomorphy of "Opisthobranchia" is the presence of a rhinophoral nerve with a thickened basis (i.e. rhinophoral ganglion) and of associated sensory structures such as Hancock's organ [66]. Based on our phylogenetic hypothesis the presence of a rhinophoral nerve has to be considered as a plesiomorphic character within Euthyneura, and thus for Panpulmonata. The rhinophoral ganglion, and potentially the optic ganglion, is considered as homologous with the pulmonate procerebrum. Rhinophoral nerve and Hancock's organ have been reduced various times independently, probably correlated with the reduction of the rhinophores and/or habitat changes.

In summary, we are currently unable to find clear morphological synapomorphies which support a placement of Acochlidia within pulmonate taxa, as sister to Eupulmonata. In the light of our phylogenetic hypothesis, conventional pulmonate synapomorphies appear to be plesiomorphies or convergences within pulmonate taxa. On the other hand, no morphological characters currently contradict that molecular phylogenetic hypothesis, nor do they favour any alternative relationships, since morphological characters common to the mesopsammic heterobranchs are shown to be convergent developments, and the potential synapomorphy of Acochlidia with "Opisthobranchia" has to be considered as plesiomorphic.

The aberrant morphology of Acochlidia in relation to its proposed sister groups remains problematic. In his ontological studies on the nudibranch *Aeolidiella alderi*, Tardy [62] reported an abnormal development in some larvae that leads to a visceral hump separated from the head-foot complex in juvenile stages, thereby closely resembling external morphology in Acochlidia (see fig. 20 in [62]). According to Tardy [62] these abnormal developmental forms are also known from pulmonate Stylommatophora. Progenesis is discussed as a principle in the evolution of meiofaunal taxa [67], and acochlidian morphology might have evolved by retention of the juvenile characters of an aberrant developmental form of an early pulmonate.

### Monophyly and phylogeny of Acochlidia

The monophyly of Acochlidia is well supported morphologically [20,22,24] and also backed by previous molecular studies [17,34]. Our study, which includes all valid acochlidian families except for the monotypic Tantulidae, also recovers Acochlidia as monophyletic but with low posterior probability and bootstrap support. The low bootstrap values for Acochlidia and some internal acochlidian taxa (e.g. Hedylopsacea) might be caused by their relatively early (Mesozoic) divergence times (see Figure 2): recent acochlidian taxa probably constitute but a remnant of much larger diversity in evolutionary history.

The acochlidian internal topology confirms the morphological analysis of Schrödl and Neusser [22], showing the same family relationships, but with better resolution within Microhedylacea: the genus *Pontohedyle *splits off at the basis of the Microhedylidae s.l. (including Ganitidae) with the closely related genera *Microhedyle *and *Paraganitus*. The hedylopsacean family Acochlidiidae includes the genera *Strubellia *and *Acochlidium *as proposed by Arnaud et al. [68] and Schrödl and Neusser [22]. Puzzling is the position of the enigmatic Aitengidae within Acochlidia, either as sister to Pseudunelidae and limnic Acochlidiidae (see Figure 1) or basal within Hedylopsacea (see Table 1). Aitengidae shows some of the general, but not unique, features of Acochlidia, such as the lack of a shell, reduction of mantle cavity, the praepharyngeal (circumpharyngeal) nerve ring, and the radula with a descending and ascending limb. This taxon also shares some features with limnic Acochlidiidae: the radula with a strong rhachidian tooth specialised in egg feeding, as also reported for *Strubellia *sp. [56]; the large, internal lateral eyes closely associated with the cerebral ganglia; and the presence of a foot groove and a branched digestive gland like reported for the genera *Acochlidium *and *Palliohedyle *[69,70]. On the other hand, Aitengidae lacks several acochlidian characteristics: the division of the body into head-foot complex and visceral hump; presence of 1-2 head appendages (with characteristic innervation of the rhinophores); and the ability to retract the head-foot complex into the visceral hump. However, in the absence of a separated visceral hump *A. ater *is able to retract its head under the notum. The presence of spicules is confirmed for Aitengidae sp., and the "parasites" described for *A. ater *might represent spicules instead (T. Neusser, pers. comm.). Re-examination of the doubtful "ascus" in *A. ater *is necessary; examination of Aitengidae sp. showed no true (i.e. sacoglossan-like) ascus containing old teeth, just a radula slightly bent at the end (own unpublished data). The presence of an ascus is currently accepted as a unique synapomorphy of Sacoglossa [4], and any sacoglossan relationship is clearly rejected by SplitsTree analysis (see Additional file 1) and phylogenetic analyses in the present study.

At the present stage of knowledge, molecular data suggests an inclusion of Aitengidae within Acochlidia, as sister to Pseudunelidae and Acochlidiidae. Detailed description by semithin serial sectioning and 3D reconstruction of the Aitengidae sp. used in the present study, together with focused redescription of *A. ater*, are needed as a basis to evaluating phylogenetic relationships of Acochlidia and Aitengidae in the future. This should be supported by a comprehensive molecular phylogeny of Acochlidia, including the two known species of Aitengidae.

### Evolutionary traits in Euthyneura

#### Invasion of the interstitial habitat

Our study supports earlier assumptions that invasion of the interstitial habitat has occurred various times independently within the Euthyneura [22,68,71], probably by benthic, sand-dwelling or temporarily (i.e. juvenile) mesopsammic ancestors of the nudibranch genera *Embletonia *and *Pseudovermis*, the cephalaspidean *Philinoglossa *and *Philine exigua*, the sacoglossan *Platyhedyle*, some members of the Rhodopemorpha *incertae sedis *(*Helminthope *and some *Rhodope*), and the Acochlidia [22,68]. The pulmonate genus *Smeagol *is found in gravel or pebble beaches on the undersides of stones; due to the relatively large body size in some species (e.g. up to 14 mm in *S. manneringi*[72]), it cannot be generally assigned to the meiofauna.

Major convergent adaptations to this spatially limited and unstable habitat are the worm-shaped body, loss of shell, and reduction of head appendages and pigmentation [21]. The development of subepidermal, calcareous spicules in Acochlidia, Rhodopemorpha and potentially *Platyhedyle *can also be considered as an adaptation to the interstitial habitat, probably serving to stabilise certain body parts during movements through the interstices [27], even though the occurrence of spicules is not limited to the mesopsammon. As far as is known, Acochlidia represent the most successful group of Heterobranchia in the mesopsammon concerning species diversity and abundance [27]. Key features for their success probably are an initial heterochronic miniaturisation and two different evolutionary trends towards a rapid, imprecise sperm transfer [23]. Additionally, adaptation to (temporarily) brackish waters with the development of a complex excretory system in Hedylopsacea [22,29] allows colonisation of shallow sands with freshwater impact (by groundwater or rain), overcoming limitations to deeper, truly marine sands.

#### Colonisation of freshwater and terrestrial habitats

It is undisputed and again confirmed by the present study that the "Pulmonata" have a marine origin [see e.g. [17,18]]. The hygrophilian radiation in the freshwater system is the most successful within "Pulmonata" [17], in terms of diversity and abundance, but not a unique event in pulmonate evolutionary history. Dinapoli and Klussmann-Kolb [14] already showed that the invasion of freshwater within pulmonate taxa took place at least twice, in Hygrophila and in the enigmatic *Glacidorbis*. According to our study, the colonisation of freshwater in Panpulmonata has occurred at least one more time in Acochlidia. Schrödl and Neusser [22] showed that within Acochlidia the freshwater colonisation already occurred twice independently, with a radiation of the Indo-Pacific Acochlidiidae and the single Caribbean *Tantulum elegans *(Tantulidae, not included in the present study). Thus, the development of a complex kidney within Hedylopsacea [29] as an adaptation to (temporarily) brackish water can be considered as a precursor to the invasion of limnic systems in Acochlidia. Acochlidian invasion of freshwater originated probably from a mesopsammic ancestor with temporary freshwater tolerance [32], or via a semi-terrestrial habitat as reported for Aitengidae [35]. Our study thus highlights the high diversity and flexibility of pulmonate habitats ranging from marine to temporarily brackish, permanently brackish, limnic and terrestrial environments. The still enigmatic *Aiteng ater *(Aitengidae) lives "amphibiously" and tolerates marine to brackish waters, but there are no observations of these animals truly leaving the water [35]. The species' mangrove habitat is comparable to that of representatives of, e.g., the pulmonate Onchidiidae, and is classified as marginal zones from which the transition to terrestrial habitat probably originated [17]. Similar to the limnic habitat, terrestrial environments have been colonised various times independently [53]. The present study indicates a least four independent pathways to the terrestrial habitat: in Amphiboloidea, Stylommatophora, Systellommatophora and Ellobioidea.

### Molecular clock and estimation of divergence times in Acochlidia

The use of molecular clocks to estimate divergence times is controversially debated, due to conflicting results from different studies and disparities with paleontological or archaeological data [73-76]. Criticism focuses on the major problems such as faulty calibration, impact of rate heterogeneity among lineages, and "time dependency of molecular rates" [73,75-77]. Some of the problems could be solved by the relaxed clock approach [78], and despite all pitfalls and criticism, molecular clock approaches have helped considerably to reveal the evolutionary history of life, especially when it comes to divergence times of groups with poor or no fossil record [75,76,79]. Thus, we consider it a valuable methodology to roughly estimate divergence times for tiny, sluggish gastropods for which there is no fossil record. Molecular clock dating stands and falls with the accuracy with which genetic distances can be estimated [80]; thus we consider the removal of ambiguous (i.e. potentially non-homologous) sites from the alignment as problematic. It seems common use to run the molecular clock analyses with reduced datasets (e.g. [14,81-83]), but the crucial question, how this will affect the molecular dating, has remained unaddressed. The exclusion of highly saturated positions - e.g., in some cases the 3^rd ^codon position of the COI sequence (see e.g. [84]) - can be justified by the biasing effect of saturation on the molecular clock. It can be argued that ambiguous parts of the alignment are often highly variable and might suffer from saturation, but on the other hand the exclusion of a series of non-saturated sites might result in underestimated divergence times. However, our Beast analysis of the raw, uncut dataset provided estimations of divergence times very similar to those from the Gblocks dataset (not shown). Nevertheless, we recommend to critically compare data from masked and raw alignments for molecular clock analyses, and to stay mindful of the potentially underestimating effect on divergence times.

The only molecular clock data on Heterobranchia [14] available prior to the present study suffers from unreliable calibration, which is considered as the most sensible and critical part of divergence time estimations [76]. There is no objective way to assign fossils to a certain point of a stem line in a recent phylogeny, thus the age of the fossil has to be taken as the minimum age of the split between the extant taxon it is assigned to and its sister group [80]. In [14] the fossil ages were assigned to the diversification of Heterobranchia, Acteonoidea and Omalogyridae, respectively, rather than to the splits from the corresponding sister groups, which led, e.g., to the surprising Pre- to early Cambrian split between Vetigastropoda and Apogastropoda. Our molecular clock was calibrated to the split between Caenogastropoda and Heterobranchia; thus molecular dating of this node is biased (i.e. depends directly on calibration features). However, fossil data shows two clearly different lineages by the mid-Devonian, thus indicating a pre- or early Devonian split of Apogastropoda [85,86]. According to our study euthyneuran gastropods already emerged in the Palaeozoic Permian, diverting from the "Lower Heterobranchia", but all major radiations of Euthyneura occurred in the early Mesozoic. According to paleontological data the oldest opisthobranchs appeared in the Triassic (about 220 Mya), the earliest pulmonates in the Jurassic (about 190 Mya) [85,86].

Based on their phylogenetic hypothesis from morphological data and the fossil record of cephalaspidean outgroups, Schrödl and Neusser [22] suspected a Jurassic time frame for the origin of Acochlidia. Their inferred sister group relationships are different from the present study, but the early divergence time is supported by our molecular clock approach, which places the origin of Acochlidia in the late Triassic to early Jurassic and their major diversification in the Jurassic. In the present study the Eupulmonata as sister group to Acochlidia show similar origin and diversification times, and so do the Hygrophila. Tillier et al. [46] inferred divergence times from branch lengths in a molecular distance tree (based on partial 28S sequences), indicating a similar Jurassic time frame for Eupulmonata and slightly younger for Hygrophila. This corresponds with fossil data, which reports a first occurrence in the late Jurassic (approx. 150 Mya) [46]. Based on fossils, diversification times of eupulmonate groups such as Stylommatophora can be dated to the late Cretaceous, when most extant families appear [87].

According to our data most acochlidian families appeared in the Jurassic or Cretaceous, only Ganitidae, Pseudunelidae and Acochlidiidae have a Palaeogene origin. These old splits on the family and even genus levels (see *Hedylopsis*, Figure 2, diverging in the Cretaceous) might indicate either that the extant diversity of Acochlidia is only a small remnant of high diversity in former times, or that known acochlidian diversity is just the tip of the iceberg still waiting to be discovered.

Based on fossil data the major diversification of "opisthobranch" taxa in a traditional sense took place comparatively recently, at the beginning of the Cenozoic (around 60 Mya), with the first records of Sacoglossa, Anaspidea and Thecosomata [86]. However, due to more or less reduced shells the fossilization probability is low. Our study suggests that most extant "opisthobranch" taxa, e.g. Sacoglossa, Cephalaspidea s.s., Pteropoda, Umbraculoidea and Anaspidea, have a Mesozoic origin. Ambiguous is the basal euthyneuran position of the Nudipleura and the resulting estimates of an old age (late Palaeozoic) and diversification (middle Mesozoic). This contradicts previous molecular clock analyses on Nudipleura, which indicated a Triassic origin and Jurassic diversification [82]. These discrepancies clearly result from major differences in tree topology (basal vs. derived position). Moreover, while our study includes only three nudipleuran representatives (poor ingroup taxon sampling), Göbbeler's and Klussmann-Kolb's [82] analysis lacks comprehensive heterobranch outgroup sampling. Future studies are needed to resolve the origin of Nudipleura within the Heterobranchia.

## Conclusions

Our multi-locus molecular study including six out of seven acochlidian families and the recently established Aitengidae confirms a pulmonate relationship of Acochlidia, which was traditionally placed within Opisthobranchia. The enigmatic Aitengidae cluster within Acochlidia. Previously assumed morphological synapomorphies of Pulmonata (pallial cavity with pneumostome, procerebrum with cerebral gland, and presence of medio-dorsal bodies) appear as either homoplastic or plesiomorphic in light of the present phylogenetic hypothesis, as does the potential opisthobranch synapomorphy (presence of rhinophoral nerve). At present, morphological characters neither justify a placement of Acochlidia within Pulmonata, nor do they favour any opisthobranch relationships that would contradict the molecular hypothesis. The aberrant acochlidian morphology might have resulted from ancestral progenesis and paedomorphic retention of the morphology of an abnormally developed juvenile.

The present study once more underlines the respective non-monophyly of Euthyneura, Opisthobranchia and Pulmonata as defined traditionally. We demonstrate the necessity for inclusion of small, enigmatic groups to solve deep-level phylogenetic relationships, and highlight that the "pulmonate" and "opisthobranch" phylogenies cannot be solved independently from each other. Clarification of remaining enigmas such as Rhodopemorpha, and of well supported taxa with unclear relationships such as Pyramidelloidea or Sacoglossa, is needed for future advances. The reclassification suggested herein defines 1) Euthyneura as including Pyramidelloidea and Glacidorboidea; 2) Euopisthobranchia as including Umbraculoidea, Cephalaspidea s.s., Runcinacea, Anaspidea and Pteropoda, but excluding Acteonoidea and Nudipleura, as well as Sacoglossa and Acochlidia; and 3) Panpulmonata as composed of Siphonarioidea, Sacoglossa, Hygrophila, Amphiboloidea, Pyramidelloidea, Glacidorboidea, Eupulmonata and Acochlidia. The present results based on standard molecular markers require confirmation from other character sets (e.g. rare genomic changes, mitochondrial gene arrangements, additional molecular markers) and careful (re-)examination of morphological characters and homology assumptions in the light of the new phylogenetic hypothesis. Our molecular clock analysis estimates a Mesozoic origin for all major panpulmonate taxa. The poorly supported topology within Panpulmonata might be promoted by the old age of this group, which potentially stands for a series of radiation and extinction events in history, resulting in poor taxon representation in present times.

The present study shows that the mesopsammon was colonised various times independently within Euthyneura, resulting in a series of convergent adaptations to the interstitial habitat. The inclusion of Acochlidia within pulmonate taxa extends the structural and biological diversity of the pulmonate clade, which exhibits remarkable flexibility in habitat choice, with various transitions from marine to limnic and terrestrial habitats.

## Methods

### Taxon sampling

A total of 78 gastropod taxa were investigated in the present study. As new material, nine acochlidian taxa and five additional enigmatic and hard-to-obtain euthyneuran taxa with potential acochlidian relationships were included (see Table 2). Specimens were collected by hand or extracted from sand samples following the method described by Schrödl [88], usually anaesthetised with MgCl_2_, and fixed in 96% ethanol. Reference specimens and DNA vouchers of sequences generated in this study are deposited at the Bavarian State Collection for Zoology (ZSM); sampling localities, reference material and DNA Bank accession numbers (http://www.dnabank-network.org) of our own data are listed in Table 2. Other sequences were retrieved from GenBank (for accession numbers see Table 3). Outgroups were chosen to include all major euthyneuran and several further heterobranch taxa. Special focus was given to mesopsammic representatives and groups previously discussed as potentially related to Acochlidia. Of these potential relatives only Rhodopemorpha are missing in our study, but a Rhodopemorpha-Acochlidia relationship can be clearly rejected based on molecular markers [49].

**Table 2:** Information on the material generated for the present study

Taxon	Family	Locality	Museums Nr.	DNA Bank voucher Nr.
**Acochlidia**				
Hedylopsis spiculifera	Hedylopsidae	Istria Croatia/Corse France, Mediterranean Sea	ZSM Mol 20080951/ZSM Mol 20080955	AB35081816 AB35081817
Hedylopsis ballantinei	Hedylopsidae	Sinai, Egypt, Red Sea	ZSM Mol 20090244	AB34858170
Pseudunela sp.	Pseudunelidae	Mounparap Island, Vanuatu, Pacific	ZSM Mol 20080393	AB35081809
Strubellia paradoxa	Acochlidiidae	Ambon, Indonesia, Indo-Pacific	Berlin Moll 193944	AB34858174
Acochlidium fijiense	Acochlidiidae	Vitilevu, Fiji, Pacific	ZSM Mol 20080063	AB34404244
Asperspina sp.	Asperspinidae	Kamtschatka, Russia, North Pacific	ZSM Mol 20090171	AB35081833
Microhedyle glandulifera	Microhedylidae	Istria, Croatia, Mediterranean Sea	ZSM Mol 20081019	AB35081799
Pontohedyle milaschewitchii	Microhedylidae	Istria. Croatia, Mediterranean Sea	ZSM Mol 20080054/ZSM Mol 20080925	AB34404241
Paraganitus ellynnae	Ganitidae	Guadalcanal, Solomons, Pacific	ZSM Mol 20080170	AB34404203
**Sacoglossa**				
Gascoignella nukuli	Platyhedylidae	Pak Phanang Bay, Thailand, Gulf of Thailand	ZSM Mol 20090182	AB344011928
Volvatella viridis	Volvatellidae	Bonotsu, Kagoshima, Japan, Pacific	-	-
Aitengidae sp.	Aitengidae	Hisamatsu, Miyako Island, Okinawa, Japan, Pacific	-	-
**Cephalaspidea**				
Philine exigua	Philinidae	Guadalcanal, Solomons, Pacific	ZSM Mol 20080752	AB34401927
Philinoglossa praelongata	Philinoglossidae	Istria, Croatia, Mediterranean Sea	ZSM Mol 20080917	AB34500041

The table lists the species names, collecting localities, reference numbers of museum vouchers (ZSM = Bavarian State Collection for Zoology; Berlin = Museum of Natural History, Berlin) and DNA vouchers deposited in the DNA Bank of the ZSM.

**Table 3:** GenBank accession numbers of the sequences used in the present study

Taxon	Family	Species	18S	28S	16S	COI
Caenogastropoda	Cyclophoridae	*Aperostoma palmeri*	DQ093435	DQ279983	DQ093479	DQ093523
	Littorinidae	*Littorina littorea*	X91970	AJ488672	DQ093481	AY345020
"Lower" Heterobranchia	Orbitestellidae	*Orbitestella *sp.	EF489352	EF489377	EF489333	EF489397
	Valvatidae	*Valvata piscinalis*	FJ917223/FJ917222	FJ917224	FJ917248	FJ917267
	Cimidae	*Cima *sp.	FJ917206.1	FJ917228.1	FJ917260.1	FJ917279.1
	Rissoellidae	*Rissoella rissoaformis*	FJ917214.1	FJ917226.1	FJ917252.1	FJ917271.1
	Pyramidellidae	*Turbonilla *sp.	EF489351	EF489376	EF489332	EF489396
	Pyramidellidae	*Boonea seminuda*	AY145367	AY145395	AF355163	-
	Pyramidellidae	*Eulimella ventricosa*	FJ917213.1	FJ917235.1	FJ917255.1	FJ917274.1
	Pyramidellidae	*Odostomia *sp.	AY427526.1	AY427491.1	FJ917256.1	FJ917275.1
	Glacidorbidae	*Glacidorbis rusticus*	FJ917211.1	FJ917227.1	FJ917264.1	FJ917284.1
Acteonoidea	Acteonidae	*Pupa solidula*	AY427516	AY427481	EF489319	DQ238006
	Aplustridae	*Hydatina physis*	AY427515	AY427480	EF489320	GQ845174.1
	Acteonidae	*Rictaxis punctocaelatus*	EF489346	EF489370	EF489318	EF489393
Nudipleura	Bathydorididae	*Bathydoris clavigera*	AY165754	AY427444	AF249222	AF249808
	Pleurobranchidae	*Tomthompsonia antarctica*	AY427492	AY427452	EF489330	DQ237992
	Pleurobranchidae	*Pleurobranchus peroni*	AY427494	AY427455	EF489331	DQ237993
Umbraculoidea	Umbraculidae	*Umbraculum umbraculum*	AY165753	AY427457	EF489322	DQ256200
	Tylodinidae	*Tylodina perversa*	AY427496	AY427458	-	AF249809
Anaspidea	Akeridae	*Akera bullata*	AY427502	AY427466	AF156127	AF156143
	Aplysiidae	*Aplysia californica*	AY039804	AY026366	AF192295	AF077759
Pteropoda	Pneumodermatidae	*Pneumoderma cf. atlantica*	DQ237970	DQ237989	-	DQ238003
	Pneumodermatidae	*Spongiobranchaea australis*	DQ237969	DQ237988	-	DQ238002
	Cavoliniidae	*Hyalocylis striata*	DQ237966	DQ237985	-	-
	Cavoliniidae	*Cavolinia uncinata*	DQ237964	DQ237983	-	DQ237997
Runcinacea	Runcinidae	*Runcina africana*	DQ923473	DQ927240	-	DQ974680
Cephalaspidea s.s.	Bullidae	*Bulla striata*	DQ923472.1	DQ986694.1	DQ986632.1	DQ986567.1
	Phillinoglossidae	*Philinoglossa praelongata*	AY427510	AY427475	HQ168411*	-
	Scaphandridae	*Scaphander lignarius*	EF489348	EF489372	EF489324	-
	Haminoeidae	*Haminoea hydatis*	AY427504	AY427468	EF489323	DQ238004
	Philinidae	*Philine exigua*	HQ168425*	HQ168438*	HQ168412*	HQ168450*
	Diaphanidae	*Diaphana *sp.	-	EF489373	EF489325	EF489394
	Diaphanidae	*Toledonia globosa*	EF489350	EF489375	EF489327	EF489395
	Cylichnidae	*Cylichna gelida*	EF489349	EF489374	EF489326	-
Sacoglossa	Volvatellidae	*Volvatella viridis*	HQ168426*	HQ168439*	HQ168413*	HQ168451*
	Cylindrobullidae	*Cylindrobulla beauii*	EF489347	EF489371	EF489321	-
	Platyhedylidae	*Gascoignella nukuli*	HQ168427*	HQ168440*	HQ168414*	HQ168452*
	Caliphyllidae	*Cyerce nigricans*	AY427500	AY427463	EU140843	DQ237995
	Plakobranchidae	*Plakobranchus ocellatus*	AY427497	AY427459	DQ480204	DQ237996
	Elysiidae	*Thuridilla bayeri*	AF249220	AY427461	DQ480206	DQ471271
	Elysiidae	*Elysia viridis*	AY427499	AY427462	AY223398	DQ237994
Sacoglossa (?)	Aitengidae	*Aitengidae *sp.	HQ168428*	HQ168441*	HQ168415*	HQ168453*
Acochlidia	Hedylopsidae	*Hedylopsis ballantinei*	HQ168429*	HQ168442*	HQ168416*	HQ168454*
	Hedylopsidae	*Hedylopsis spiculifera*	HQ168430*	HQ168443*	HQ168417*	HQ168455*
	Pseudunelidae	*Pseudunela *sp.	HQ168431*	HQ168444*	HQ168418*	HQ168456*
	Acochlidiidae	*Strubellia paradoxa*	HQ168432*	HQ168445*	HQ168419*	HQ168457*
	Acochlidiidae	*Acochlidium fijiense*	HQ168433*	HQ168446*	HQ168420*	HQ168458*
	Asperspinidae	*Asperspina *sp.	HQ168434*	HQ168447*	HQ168421*	-
	Microhedylidae	*Pontohedyle milaschewitchii*	HQ168435*	AY427484	HQ168422*	HQ168459*
	Ganitidae	*Paraganitus ellynnae*	HQ168436*	HQ168448*	HQ168423*	HQ168460*
	Microhedylidae	*Microhedyle glandulifera*	HQ168437*	HQ168449*	HQ168424*	HQ168461*
Siphonarioidea	Siphonaridae	*Siphonaria pectinata*	U86321	DQ279993	AY377627	AF120638
	Siphonaridae	*Siphonaria concinna*	EF489334	EF489353	EF489300	EF489378
Amphiboloidea	Amphibolidae	*Amphibola crenata*	EF489337	EF489356	EF489304	-
	Amphibolidae	*Phallomedusa solida*	DQ093440	DQ279991	DQ093484	DQ093528
	Amphibolidae	*Salinator cf. fragilis*	-	EF489355	EF489303	EF489381
Hygrophila	Latiidae	*Latia neritoides*	EF489339	EF489359	EF489307	EF489384
	Chilinidae	*Chilina *sp.	EF489338	EF489357	EF489305	EF489382
	Acroloxidae	*Acroloxus lacustris*	AY282592	EF489364	EF489311	AY282581
	Lymnaeidae	*Lymnaea stagnalis*	EF489345	EF489367	EF489314	EF489390
	Physidae	*Physella acuta*	AY282600	EF489368	AY651241	AY282589
	Planorbidae	*Ancylus fluviatilis*	AY282593	EF489365	EF489312	AY282582
Stylommatophora	Arionidae	*Arion silvaticus*	AY145365	AY145392	AY947380	AY987918
	Helicidae	*Arianta arbustorum*	AY546383	AY014136	AY546343	AY546263
	Enidae	*Ena montana*	AY546396	-	AY546356	AY546276
	Cerionidae	*Cerion incanum*	-	AY014060.1	-	-
	Subulinidae	*Rumina decollata*	-	13794085:464-1292	AY345050	AY345050
Systellommatophora	Onchidiidae	*Onchidium verruculatum (§)*	AY427522	AY427487	EF489316	EF489391
	Onchidiidae	*Onchidella floridiana*	AY427521	AY427486	EF489317	EF489392
	Veronicellidae	*Laevicaulis alte*	X94270.1	AY014151.1		
	Veronicellidae	*Semperula wallacei*	-	DQ897671.1	DQ897675.1	DQ897673.1
	Rathouisiidae	*Atopos australis*	-	AY014152.1	-	-
Trimusculoidea	Trimusculidae	*Trimusculus afra*	EF489343	-	EF489309	EF489388
Otinoidea	Otinidae	*Otina ovata*	EF489344	EF489363	EF489310	EF489389
	Smeagolidae	*Smeagol phillipensis*	FJ917210	FJ917229	FJ917263	FJ917283
Ellobioidea	Carychiidae	*Carychium minimum*	EF489341	EF489361	EF489308	EF489386
	Ellobiidae	*Ophicardelus ornatus*	DQ093442	DQ279994	DQ093486	DQ093486
	Ellobiidae	*Myosotella myosotis*	EF489340	EF489360	AY345053	EF489385

Sequences generated within this study are marked with *; (§) in GenBank as "*O. verrucosum*", which is not a valid name, thus treated as *O. verruculatum. *(" - " indicates missing sequences).

### DNA extraction, PCR and sequencing

Genomic DNA was extracted from tissue samples of the foot or from entire specimens using the DNeasy Blood and Tissue Kit (Qiagen, Hilden Germany). Four markers were amplified: nuclear 18S rRNA (approx. 1800 bp), 28S rRNA (approx. 1020 bp), mitochondrial 16S rRNA (approx. 300-400 bp), and cytochrome *c *oxidase subunit I (COI - approx. 650 bp). For PCR protocols and primers used, see additional file 3. Successfully amplified PCR products were purified using ExoSapIT (USB, Affymetrix, Inc.). Cycle sequencing and the sequencing reaction were performed by the sequencing service of the Department of Biology Genomic Service Unit (GSU) of the Ludwig-Maximilians-University Munich, using Big Dye 3.1 kit and an ABI 3730 capillary sequencer. All fragments were sequenced in both directions using the PCR primers. All sequences have been deposited at GenBank (see Table 3 for accession numbers). The Gblock alignment and the resulting tree were deposited in TreeBASE (http://www.treebase.org, accession number 10801).

### Sequence editing and alignment

All sequences generated in this study were checked for contaminations with BLAST searches [89] implemented in the GenBank database on the NCBI webpage (http://blast.ncbi.nlm.nih.gov/Blast.cgi). Reconciliation of forward and reverse reads was carried out in BioEdit 7.0.5. [90]. MAFFT v6 [91] was used to generate sequence alignments for each gene region, using the default settings (automatically chosen models for 18S, 28S, COI: FFT-NS-i; for 16S: L-INS-i). The alignment of the protein coding COI gene was corrected manually according to the amino acids. The individual MAFFT alignments were parsed 1) using Gblocks [92,93] with the default settings for less stringent selection, 2) with ALISCORE v1.0 [94] using the default parameters, or c) left unmasked.

### Phylogenetic analysis

For an *a priori *analysis of variation in the phylogenetic signal a split-decomposition analysis was performed using SplitsTree v4.6 [95].

The best-fit model of nucleotide substitution for each gene was selected using Modeltest 3.7 [96] via the Akaike Information Criterion (AIC). The incongruence length difference (ILD) test [97] was carried out in Paup 4.0b10 [36]. This test was conducted with heuristic searches and 100 replicates to evaluate incongruence between single markers.

Maximum likelihood analyses were performed using RAxML 7.0.3 [98] adapting the program parameters to the alignment as described in the manual ("hard & slow way" - with 10 parsimony starting trees and 6 different rate categories). Additionally 200 multiple inferences were executed on the original alignment and 1000 bootstrap replicates were generated. Analyses were run under the GTR Gamma model as recommended in the manual [98] and the caenogastropod taxa *Littorina littorea *and *Aperostoma palmeri *were defined as outgroups. The alignment was analysed in different partition sets: one partition, two partitions (18S + 28S + 16S combined; COI separate), three partitions (18S + 28S + 16S combined; COI with codons partitioned to 1^st ^+ 2^nd ^separate from 3^rd^), four partitions (separated by gene regions), and five partitions (18S, 28S, 16S, COI 1^st ^+ 2^nd^, COI 3^rd^). To test whether partitioning significantly improves the likelihood values of the dataset, we compared the likelihood values of all partitions via the Akaike Information Criterion.

Bayesian phylograms were generated from the Gblocks and ALISCORE alignments with MrBayes 3.1.2 [99]. The general time-reversible model was used for both datasets, with invariant site frequency and gamma-shape parameter estimated from the data (GTR + I + G). The 'shape', 'proportion of invariant sites', 'state frequency' and 'substitution rate' parameters were estimated for each gene separately. Each codon position in the amino-acid coding COI was also allowed to have different parameters; hence the alignments had six partitions of parameters. Two parallel runs were made for 5 × 10^6 ^generations (with a sample frequency of 1000), using a default value of four Markov chains. Quality and ESS values (effective sampling size) of each run were checked in Tracer 1.5.3. The first 2000 trees for each run were discarded to ensure that the four chains reached stationarity. The consensus tree and posterior probabilities were computed from the remaining 6000 trees (3000 trees × 2 runs).

To evaluate support for our tree topology an alternative topology (grouping Acochlidia with Opisthobranchia) was tested in comparison to the "best" tree topology by using the Approximately Unbiased Test [100]. The hypothetic topology was computed with RAxML [98] using the -g option for the constraint ML tree. The p-values of the sitewise log likelihoods combined with the "best" topology were estimated using Treefinder [101].

### Molecular clock

Approximate divergence times were calculated using the relaxed molecular clock approach [78] implemented in the software BEAST 1.5.3 [102]. For molecular clock analysis the concatenated Gblock-dataset was analysed in five partitions as for the phylogenetic analyses.

Calibration points were chosen for groups with stable and well supported nodes in the phylogenetic hypothesis and decently documented fossil record with clear identification to recent taxa. Minimum constraints for three nodes were chosen based on the fossil record: 1) split between Caenogastropoda and Heterobranchia based on the oldest known fossil of the Heterobranchia (*Palaeocarboninia janke*) recorded from the Middle Devonian (390 Ma) [85]; 2) the split between Acteonoidea and its sister group based on acteonoid fossils with a minimum age of 240 Ma ([103], A Nützel pers. comm.) and 3) the split of Ellobioidea and their sister group based on ellobiid fossils with a minimum age of 140 Ma ([86], A Nützel pers. comm.). We calibrated using a hard minimum bound (i.e. the divergence data cannot be younger than the oldest known fossil); the probability that the divergence event occurred above the minimum date declines according to a gamma distribution, such that 95% of the posterior density falls within the range [x - x + 10%] [see [104]]. Calibration nodes were not fixed as monophyletic.

The analyses were run with the relaxed uncorrelated lognormal clock model under the Yule process using the GTR+G+I substitution model (chosen from Modeltest 3.7 [96] via the Akaike Information Criterion) for all markers. The MCMC was run ten times independently, generating 10^6 ^generations each, and sampled every 1000 steps. The single runs were combined with LogCombiner 1.5.3, with the first 10^5 ^samples each discharged as burn-ins. The runs were checked for quality and sufficient ESS (effective sample size) in Tracer 1.5.3. All trees were combined to produce a consensus tree using TreeAnnotator 1.5.3, with the first 1000 trees of each dataset discharged as burn-in.

To evaluate the potential effect on molecular dating of removing ambiguous sites from the alignment, the BEAST runs were repeated with the raw alignments (i.e. mainly uncut; only longer ends of some sequences removed due to the use of different primers) alignments, generating 10 × 10^6 ^generations and following the method described above.

## Competing interests

The authors declare that they do not have competing interests.

## Authors' contributions

KMJ, MS, YK and HF sampled the material. KMJ, IS, TK and YK generated the molecular data. KMJ and YK conducted the phylogenetic and network analysis. KMJ performed the molecular clock approach. KMJ wrote the initial version of the manuscript; all authors contributed to the discussion of the results and the preparation of the final manuscript. MS planned and supervised the study. All authors have read and approved the final manuscript.

## Supplementary Material

Additional file 1 **Neighbournet graph on the origin of Acochlidia**. Generated with Splits Tree v4.6 from the concatenated, four marker dataset masked with Gblocks, visualising highly conflicting signal at the basis of the Acochlidia. Representatives of meiofaunal taxa highlighted in boldface, showing the absence of a common phylogenetic signal. Click here for file

Additional file 2 Likelihood values of different partitions Click here for file

Additional file 3 **PCR protocols and primers used **[105-107]. Click here for file
