# Supplementary figures and images for: On the origin of Acochlidia and other enigmatic euthyneuran gastropods, with implications for the systematics of Heterobranchia
Source: BMC Evol Biol. 2010 Oct 25;10:323. doi: 10.1186/1471-2148-10-323 (PMC3087543; doi:10.1186/1471-2148-10-323)

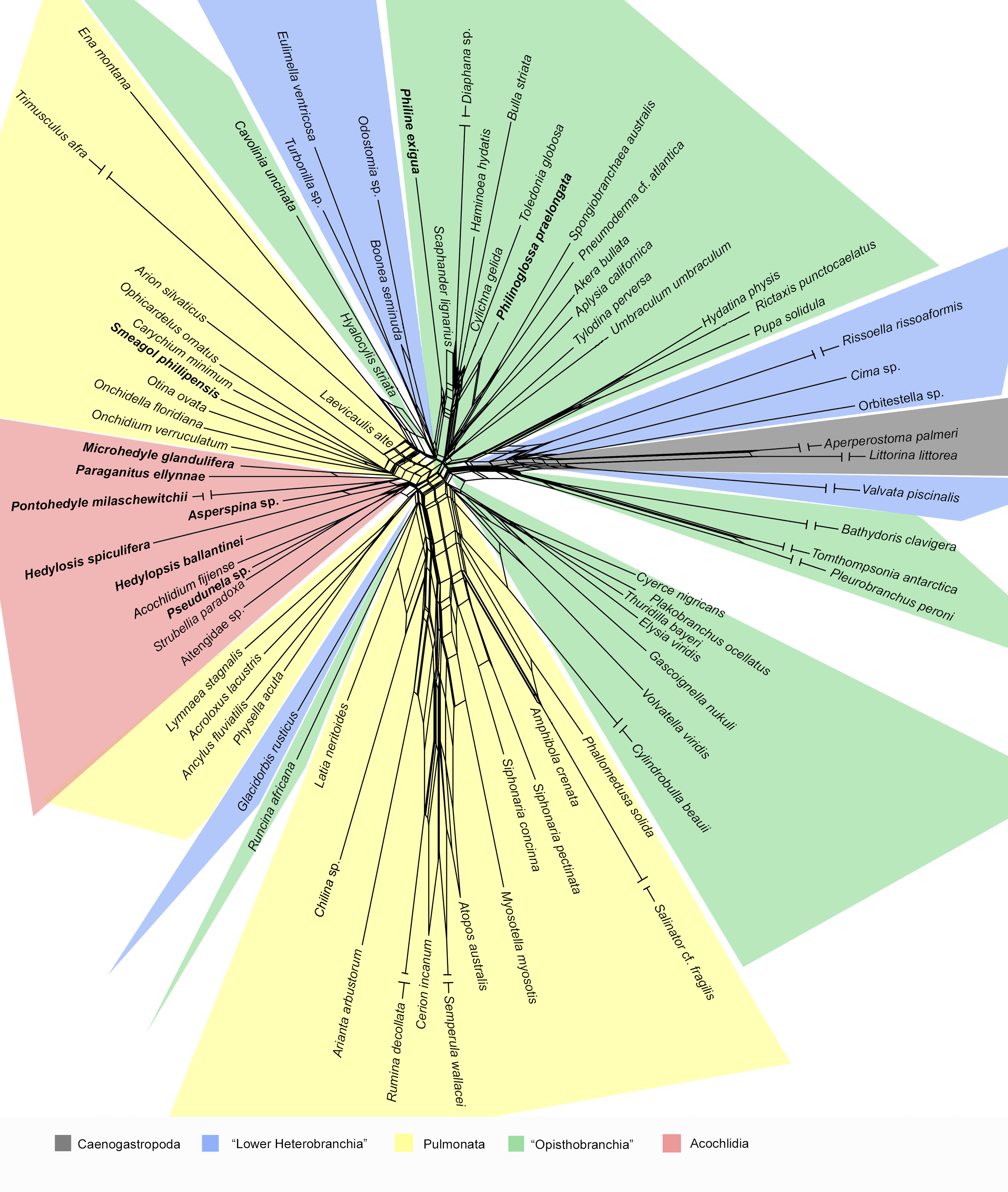

Supplement: Additional file 1 — Neighbournet graph on the origin of Acochlidia. Generated with Splits Tree v4.6 from the concatenated, four marker dataset masked with Gblocks, visualising highly conflicting signal at the basis of the Acochlidia. Representatives of meiofaunal taxa highlighted in boldface, showing the absence of a common phylogenetic signal. [file 1471-2148-10-323-S1.TIFF]
