# Supplementary material for: On the origin of Acochlidia and other enigmatic euthyneuran gastropods, with implications for the systematics of Heterobranchia
Source: BMC Evol Biol. 2010 Oct 25;10:323. doi: 10.1186/1471-2148-10-323 (PMC3087543; doi:10.1186/1471-2148-10-323)
Supplement: Additional file 2 — Likelihood values of different partitions [file 1471-2148-10-323-S2.DOCX]

| **Partitions** | **log likelihoods Gblock dataset** | **log likelihoods Aliscore dataset** |
| --- | --- | --- |
| partition 1 (entire alignment) | -61663,405401 | -67383.535212 |
| partition 2 (18S + 28S + 16S combined; COI separate) | -60696,519517 | -66463.267795 |
| partition 3 (18S + 28S + 16S combined; COI with codons partitioned to 1^st^ + 2^nd^ separate from 3^rd^) | -59587,363602 | -65346.412291 |
| partition 4 (separated by gene regions) | -59999,413744 | -65583.189501 |
| partition 5 (18S, 28S, 16S, COI 1^st^ + 2^nd^, COI 3^rd^) | -58864,842767 | -64431.909825 |
