# Supplementary material for: On the origin of Acochlidia and other enigmatic euthyneuran gastropods, with implications for the systematics of Heterobranchia
Source: BMC Evol Biol. 2010 Oct 25;10:323. doi: 10.1186/1471-2148-10-323 (PMC3087543; doi:10.1186/1471-2148-10-323)
Supplement: Additional file 3 — PCR protocols and primers used [105-107]. [file 1471-2148-10-323-S3.DOCX]

| **Gene region** | **Primer** | **Sequence 5´ - 3´** | **Reference** | **PCR program** |
| --- | --- | --- | --- | --- |
| **18S** | 18A1 | CCT ACT TCT GGT TGA TCC TGC CAG T | [105] | 98°C 30sec (98°C 5sec, 48-65°C 5sec, 72°C 20-25sec) x 28-40, 72°C 60sec  (Phire polymerase, New England Biolabs) |
|  | 700R | CGC GGC TGC TGG CAC CAG AC | [34] |  |
|  | 470F | CAG CAG GCA CGC AAA TTA CCC | [34] |  |
|  | 1500R | CAT CTA GGG CAT CAC AGA CC | [34] |  |
|  | 1155F | CTG AAA CTT AAA GGA ATT GAC GG | [34] |  |
|  | 1800 | TAA TGA TCC TTC CGC AGG TT | [105] |  |
| **28S** | 28SC1 | ACC CGC TGA ATT TAA GCA T | [12] | 98°C 30sec (98°C 5sec, 48-65°C 5sec, 72°C 20-25sec) x 28-40, 72°C 60sec  (Phire polymerase, New England Biolabs + Q-solution, Qiagen) |
|  | 28SD2R | CCT TGG TCC GTG TTT CAA GAC GGG | [34] |  |
|  | 28SC2F | GAA AAG AAC TTT GAA GAG AGA GT | [34] |  |
|  | 28SD3 | GACGAT CGA TTT GCA CGT CA | [34] |  |
| **16S** | 16S-H | CGC CTG TTT ATC AAA AAC AT | [106] | 98°C 30sec (98°C 5sec, 48-55°C 5sec, 72°C 25sec) x 35-40, 72°C 60sec  (Phire polymerase, New England Biolabs) |
|  | 16S-R | CCG GTC TGA ACT CAG ATC ACG T | [106] |  |
|  | 16Sf-50 | GGC CGC AGT ACC TTG ACT GT | present study |  |
|  | 16Sr-380 | TCC ACC ATC GAG GTC ACA AG | present study |  |
| **COI** | LCO1490 | GGT CAA CAA ATC ATA AAG ATA TTG G | [107] | 94°C 3min (94°C 60sec, 48-52°C 60sec, 72°C 90sec) x 35-40, 72°C 3min  (Taq polymerase, Sigma) |
|  | HCO2198 | TAA ACT TCA GGG TGA CCA AAA AAT CA | [107] |  |
